# Effects of Combined Peat Powder and Fermented Manure on Oat Yield, Soil Enzyme Activities and Microbial Community Composition in Horqin Sandy Land

**DOI:** 10.3390/biology15191780

**Published:** 2026-10-08

**Authors:** Ruozhuang Zhao, Haishuang Liu, Jiale Na, Tao Li, Ziwei Li, Tiexia Zhu, Zhaoxu Nie, Kai Gao

**Affiliations:** College of Grassland, Inner Mongolia Minzu University, Tongliao 028000, China

**Keywords:** sandy land, fermented manure, peat powder, microbial community, soil enzyme activities, soil nutrient

## Abstract

Low soil fertility caused by organic matter deficit severely restricts crop productivity in sandy lands. To investigate the effects of organic matter addition on plant yield and soil productivity in sandy areas, a two-factor randomized block field experiment was conducted using oat (*Avena sativa*) with five ratios (7:3, 3:2, 1:1, 2:3, 3:7; w:w) and five application rates (0, 5, 10, 15, and 20, t·ha^−1^) of peat powder and fermented manure in the Horqin Sandy Land, which is a typical agro-pastoral ecotone in northern China. Application of combined peat powder and fermented manure significantly increased oat yield, and the fertilization rate exerted a stronger effect on oat yield than the mixing ratio of the two organic materials. Furthermore, organic fertilization significantly increased the relative abundances of microorganisms, improved soil C, N and P contents, and enhanced the activities of urease, invertase, and catalase. The combination of an application rate of 20 t·ha^−1^ and a 3:2 peat-powder-to-fermented-manure ratio represents a promising fertilization regime among treatments tested for oat cultivation in this region, given its highest aboveground dry-matter yield. Collectively, these findings show that rational combined application of fermented manure and peat powder in sandy land can significantly increase soil enzyme activities and optimize soil nutrient stoichiometry, and is positively correlated with rhizosphere microbial community composition and oat yield.

## Simple Summary

Sandy farmland suffers from poor soil fertility due to insufficient organic matter, which greatly limits crop production. This study aimed to find effective fertilization methods to improve soil quality and crop yield in sandy lands. We conducted a field experiment in the typical sandy farming area of northern China, planting oats with different mixing ratios and application amounts of peat powder and fermented manure. The results confirmed that organic fertilization clearly improved oat yield, soil nutrients, soil microbial quantity and soil enzyme activity. The amount of fertilizer used had a larger impact on crop yield than the mixing ratio of the two materials. We identified the best fertilization scheme for local oat planting from all tested treatments. This study verifies that scientific mixed application of these two organic materials can effectively improve barren sandy soil and raise crop output, providing a reliable and practical planting strategy for sandy land agriculture.

## 1. Introduction

Intrinsically low soil organic matter stock constitutes the primary limiting factor restricting the sustainable development of farming and pastoral industries across sandy and desertified regions, and this phenomenon is prevalent in all sandy land areas of northern China. The Horqin Sandy Land was historically a region of lush grasslands and a major livestock production base in northern China. However, rapid population growth, excessive reclamation, overgrazing, and unsustainable land-use practices, coupled with the region’s arid climate, high evaporation rates, strong winds, and sandy soils that are highly susceptible to erosion, have led to severe soil desertification, with soil organic matter contents ranging from 0.6 to 4.57 g·kg^−1^, generally low [1,2]. Soil organic matter not only provides nutrients for microbial activity but also supplies essential elements such as C, N, and P for crop growth. Therefore, enhancing soil organic matter content is a primary approach to restoring soil fertility and realizing sustainable ecosystem development.

Organic fertilizer not only provides directly accessible nutrients to plants, but also supplies carbon and nutrient sources for soil microorganisms [3,4], thereby inducing changes in microbial diversity and community structure [5,6]. The microbial communities play a critical role during crop growth by enhancing the mineralization of organic materials and producing bioavailable compounds, supplying nutrients to plants. Soil carbon, nitrogen, and phosphorus (C:N:P) stoichiometry is fundamental to soil fertility [7], directly determining soil nutrient reserves and supply–demand efficiency. Among them, elevated soil organic carbon (SOC) content promotes the formation of stable soil structures, which not only supply nutrients for plant growth but also improve soil porosity and water performance capacity [8]. Nitrogen and phosphorus can rapidly enhance soil fertility and play a vital role in aboveground plant growth, development, and ecosystem productivity [9]. Previous studies have shown that continuous organic fertilizer application significantly increases soil SOC and TN contents.

Fermented manure and peat powder are high-quality raw materials for organic fertilizers. Fermented manure is rich in organic matter and various micronutrients, capable of substantially increasing soil organic matter content [10,11]. Peat powder can rapidly supplement soil organic carbon stocks and increase soil porosity, thereby enhancing soil water-holding and nutrient-retention capacities [12,13], making it a major strategy for improving soil fertility in sandy land. The application of organic fertilizers can effectively improve soil fertility and promote forage productivity [14,15]. However, most existing studies have focused primarily on the ameliorative effects of a single manure type on soil. Previous studies have demonstrated that single organic amendments can effectively improve sandy soil properties [16]. However, sole application of sheep manure in sandy farmlands easily causes rapid nutrient release and severe nutrient leaching, limiting its fertilization sustainability [17]. In contrast, peat powder can effectively improve soil physical and chemical structure but cannot provide sufficient available nutrients to support optimal crop productivity [13]. Although the individual effects of these two organic materials are well documented, their combined application may produce synergistic effects that differ from single application. However, the regulatory mechanisms by which the combined application of fermented sheep manure and peat powder impacts soil microbial communities, enzyme activities, and physicochemical properties in sandy farmlands remain unclear. Clarifying these interactive responses is essential for optimizing organic fertilization strategies for sandy soil improvement. We hypothesized that: (1) different application rates and ratios of organic fertilizer significantly affect oat yield and (2) changes in microbial community composition are associated with organic amendments and soil physicochemical properties, and these soil traits are significantly correlated with crop aboveground yield.

Therefore, the present study established oat (*Avena sativa*) cultivation grasslands in the Horqin sandy farmlands as the research subject and conducted field experiments with various ratios and application rates of fermented sheep manure and peat powder. The objectives were to elucidate the effects of organic amendment on soil C, N, and P stoichiometric characteristics, enzyme activities, and microbial diversity in the Horqin Sandy Land, and to reveal the underlying mechanisms by which organic fertilization enhances oat productivity. This study aims to provide a theoretical basis and technical guidance for improving the supply of high-quality forage in sandy farmlands.

## 2. Materials and Methods

### 2.1. Study Site

The experimental site is located in the Science and Technology Demonstration Park of Inner Mongolia University for Nationalities (43°3′ N, 122°2′ E), with an altitude of approximately 178 m. The experimental soil is aeolian sandy soil, with pH 8.2, soil organic matter of 4.86 g kg^−1^, available phosphorus of 10.56 mg kg^−1^, available nitrogen of 11.15 mg kg^−1^, and available potassium of 94.65 mg kg^−1^. In 2024, the annual average temperature was 7.92 °C, the annual average maximum temperature was 14.52 °C, and the annual average minimum temperature was 1.61 °C. The annual sunshine duration reached 3399.15 h, and the annual average relative humidity was 59.16%. During the oat growing season (May–September) of 2024, the mean temperature was 18.1 °C, and the total precipitation during this period was 316 mm, with precipitation concentrated in summer.

### 2.2. Experimental Materials

The crop tested in this study was oat cultivar ‘Nelson’. The commercial organic fertilizer used in this experiment was prepared using peat (organic matter ≥ 50%, pH 5.5–6.5, total N-P-K content < 2%) and sheep manure (organic matter ≥ 30%, N + P_2_O_5_ + K_2_O ≥ 4%). Peat powder was obtained by air-drying peat, grinding it, and sieving through a 2 mm mesh, yielding peat powder with particle size < 2 mm. Fermented sheep manure was produced by mixing raw sheep manure, crushed straw, water, and microbial inoculants for composting. The moisture content was maintained at 50–60%. The compost inoculant was second-diluted at a ratio of 1 kg inoculant to 15 kg fine dry substrate before thorough incorporation into the mixture. The compost pile was stacked to a height of 1–1.5 m, with pH maintained within 5–8. The first turning was performed when pile temperature exceeded 50 °C, followed by turning every 2 d for a total of eight turning events to complete composting.

### 2.3. Experimental Design

#### 2.3.1. Experimental Setup

A two-factor randomized block design was employed in this field experiment. The experimental treatments consisted of two variables: organic fertilizer proportion and fertilization rate. The organic fertilizer proportion and fertilization rate, and their interaction, were treated as fixed effects; block was treated as a random effect.

Organic fertilizer proportion (D levels):

Five mixing ratios of peat powder and fermented sheep manure were established (w:w), including 70% peat powder + 30% sheep manure (D1), 60% peat powder + 40% sheep manure (D2), 50% peat powder + 50% sheep manure (D3), 40% peat powder + 60% sheep manure (D4), and 30% peat powder + 70% sheep manure (D5).

Fertilization rate (A levels):

Five fertilization gradients were set as 0 t ha^−1^ (A1), 5 t ha^−1^ (A2), 10 t ha^−1^ (A3), 15 t ha^−1^ (A4), and 20 t ha^−1^ (A5).

In total, 25 combined treatments were generated in this experiment. Each treatment under different organic fertilizer proportion and fertilization rate was performed with four biological replicates, resulting in 100 experimental plots. The area of each individual plot was 20 m^2^ (Figure S1).

#### 2.3.2. Artificial Grassland Establishment and Fertilization Management

The artificial oat grassland was established on 1 May 2024. Prior to sowing, compound fertilizer (40 kg N ha^−1^, 26.18 kg P ha^−1^, 49.81 kg K ha^−1^) was uniformly applied at a rate of 250 kg ha^−1^ as base fertilizer and thoroughly incorporated into the top 0–20 cm soil layer of each plot. All organic fertilizer amendments were uniformly incorporated into the topsoil before oat strip sowing. The oat sowing rate was set at 105 kg ha^−1^, with a row spacing of 0.2 m and a soil covering depth of 2 cm after sowing.

### 2.4. Aboveground Dry-Matter Yield Determination and Soil Sample Collection

#### 2.4.1. Aboveground Dry-Matter Yield Determination

During the oat filling stage, a 1 m^2^ quadrat was randomly selected for sampling in each plot. All plant samples were harvested and weighed using the gravimetric method. The samples of fresh forage were dried at 105 °C for 30 min followed by 65 °C for 48 h, re-dried, and re-weighed, until a constant weight was achieved. Finally, the aboveground dry-matter yield of each plot was calculated and converted to a uniform unit area.

#### 2.4.2. Soil Sample Collection

Soil samples were collected from the 0–20 cm soil layer. For rhizosphere soil sampling, 10 healthy oat plants were dug out in each plot. After shaking off loosely attached bulk soil, the soil tightly adhering to root surfaces was collected as rhizosphere soil. The approximate weight of each composite rhizosphere soil sample was 20 g.

The five-point sampling method and root shaking method were adopted to collect bulk soil and rhizosphere soil samples. All rhizosphere and bulk soil samples were collected in pairs from each plot (one rhizosphere sample paired with one bulk soil sample per plot). The experimental unit was each field plot, while observational units corresponded to individual rhizosphere and bulk soil samples collected from each plot. The collected soil samples were divided into two parts. One portion was stored fresh at low temperature for the determination of soil microbial community structure and soil enzyme activities. The other portion was naturally air-dried in a cool and ventilated place and sieved for the subsequent determination of soil physicochemical properties [18].

### 2.5. Measurement Indicators and Methods

Urease activity was determined using the sodium phenate–sodium hypochlorite colorimetric method [19]. Invertase activity was assayed by the 3,5-dinitrosalicylic acid colorimetric method [20]. Alkaline phosphatase activity was measured using the disodium phenyl phosphate colorimetric method [21]. Catalase activity was determined by potassium permanganate titration [22]. Soil organic carbon (SOC) content was analyzed using the potassium dichromate external heating method [23]. Total nitrogen (TN) content was determined by the Kjeldahl method following H_2_SO_4_–H_2_O_2_ digestion [23]. Total phosphorus (TP) content was measured using the molybdenum–antimony–ascorbic acid colorimetric method [23]. The carbon-to-nitrogen ratio (C:N) was calculated as the ratio of SOC to TN, the carbon-to-phosphorus ratio (C:P) as the ratio of SOC to TP, and the nitrogen-to-phosphorus ratio (N:P) as the ratio of TN to TP. The C:N:P stoichiometric ratios are mass-based ratios.

Soil microbial community composition was characterized by amplicon sequencing of the 16S rRNA gene for bacteria and the internal transcribed spacer (ITS) region for fungi [24]. In brief, soil DNA was extracted from 0.5 g (wet weight) of each soil sample using the ISOIL for Beads Beating Kit (Nippon Gene, Tokyo, Japan) according to the manufacturer’s protocol. The concentration and purity of DNA were detected using a NanoDrop D-1000 spectrophotometer (NanoDrop Technologies, Wilmington, DE, USA). Amplicon sequencing of soil bacterial 16S rRNA and fungal ITS regions was performed on a DNBSEQ-T7 instrument (MGI-Tech, Shenzhen, China). The V3-4 hypervariable region of the bacterial 16S rRNA gene was amplified using the universal primers 338F (5′-ACTCCTACGGGAGGCAGCAG-3′) and 806R (5′-GGACTACNNGGGTATCTAAT-3′). Primers for fungal ITS were *its1* (5′-CTTGGTCATTTAGAGGAAGTAA-3′) and *its2* (5′-GCTGCGTTCTTCATCGATGC-3′). The Benjamini–Hochberg FDR correction (Q < 0.05) was used for multiple-testing adjustment when analyzing large sets of microbial taxa. Paired-end clean reads were assembled using FLASH software (v1.2.11). Chimeric sequences were identified and removed using the UCHIME algorithm. The valid sequences were clustered into amplicon sequence variants (ASVs) at a 97% similarity threshold using UPARSE. Taxonomic classification of bacterial 16S rRNA sequences was performed against the Silva database (version 138). Fungal ITS sequences were annotated using the UNITE database (version 8.2). Model-fit indices including comparative-fit index (CFI), Tucker–Lewis index (TLI), root-mean-square error of approximation (RMSEA) with its 90% confidence interval, and standardized root-mean-square residual (SRMR) were reported for model evaluation. Model acceptance criteria: CFI and TLI > 0.90, RMSEA ≤ 0.08, and SRMR ≤ 0.08.

### 2.6. Data Analysis

Excel 2019 was used for preliminary data sorting and basic statistical analysis, including the calculation of mean values and standard error (SE) of each measured index. QIIME2-2025.4 software was applied to calculate soil microbial alpha diversity indices. Python (v3.9.15) scripts were used to analyze the taxonomic composition of bacterial and fungal communities at the genus level and identify dominant microbial phyla and genera. Linear discriminant analysis effect size (LEfSe) was performed to identify differentially abundant taxa across treatments, based on the genus-level relative-abundance matrix generated by BGI-Shenzhen following amplicon sequencing. The Kruskal–Wallis test was used to detect taxa with significant inter-group differences (*p* < 0.05), followed by pairwise Wilcoxon rank-sum tests for sub-group comparisons (*p* < 0.05). The LDA score threshold was set to 2.0, and all other parameters adopted the default settings of the analysis platform. Random forest regression models were constructed to screen genera showing explanatory associations with oat yield. Structural equation modeling (SEM) was further used to reveal the multi-level regulatory relationships among organic fertilizer application, microbial communities, soil extracellular enzyme activities, soil nutrient contents, and oat yield. IBM SPSS Statistics 27 was used for two-way analysis of variance (ANOVA). Duncan’s multiple range test was applied to determine the significant differences among treatments. All figures and graphs were plotted using Origin 2024 software.

## 3. Results

### 3.1. Effects of Organic Fertilizer Application on Oat Yield

High yield is a core objective of agricultural and animal husbandry production. In terms of fertilization rate, an increase in the amount of fertilizer applied significantly enhanced the yield, except for the A5 treatment under the D4 and D5 conditions (Figure 1). Compared to A4, no significant increase in yield was detected for treatment A5 under D4, and yield was significantly reduced in A5 under D5 to 8.39 t ha^−1^ (Figure 1). As for fertilizer proportion, treatment D5 had the highest yield under treatments A2-A4; by contrast, under the A5 condition, yields reached 7.57, 8.16 and 9.07 t ha^−1^. The highest yield was observed under treatment D2 (Figure 1). Among all combinations, treatment D2A5 produced the highest yield of 9.26 t ha^−1^ (Figure 1). Among all tested treatments, the combination of 20 t·ha^−1^ application rate with a peat-powder-to-fermented-manure ratio of 3:2 exhibited the greatest aboveground dry-matter yield, showing good application potential for local oat production.

### 3.2. Effects of Organic Fertilizer Addition on Fungal Communities

Fertilization rate exerted a highly statistically significant effect on the relative abundance of *Pseudogymnoascus* in rhizosphere soil, as well as *Alternaria* and *Bipolaris* in bulk soil (*p* < 0.01) (Table 1). In rhizosphere soil, the relative abundance of *Alternaria* was the highest in D2A2 (22.93%), and *Bipolaris* attained its maximum value of 21.58% under D5A2 (Table 1). The highest relative abundance of *Fusarium* (18.67%) and *Pseudogymnoascus* (6.46%) occurred in D5A5 and D3A5, respectively (Table 1). In bulk soil, the relative abundance of *Fusarium* peaked under treatment D2A4 at 20.88%, while *Pseudogymnoascus* reached its maximum relative abundance of 10.21% in treatment D5A4 (Table 1). The highest relative abundances of *Alternaria* (10.43%) and *Bipolaris* (7.45%) were both observed in D2A1 (Table 1). It can be seen that organic fertilization altered the compositional structure of soil fungal communities. The relative abundance of *Pseudogymnoascus* and *Fusarium* increased with rising fertilization rates under certain mixing-ratio conditions, whereas *Alternaria* and *Bipolaris* generally decreased as fertilization rate increased.

Organic fertilizer proportion exerted a significant effect on the fungal Shannon index in bulk soil (*p* < 0.05) (Figure S2A). Under condition A, the Shannon index under treatment D3 was significantly higher than those of D1, D2, and D5 in rhizosphere soil (Figure S2A). Meanwhile, under A4 and A5, the highest Shannon index was observed in D1, which was significantly higher than that of D2 (Figure S2B). The fungal Shannon index of bulk soil was significantly higher than that of rhizosphere soil, with an increasing range of 0.58–34.7% (Figure S2C,D).

For fertilization rate, no biomarker taxa with significant differences were observed at both phylum and class levels (Figure S2A,B). At the genus level, *Cenococcum*, *Pleotrichocladium*, *Polyschema*, *Bulleribasidium*, *Paradevriesia*, *Dlhawksworthia*, *Neocucurbitaria* and *Redeckera* were significantly enriched in A1, whereas *Acaulium* was enriched in A4. *Truncatella* and *Blsatomyces* showed significant enrichment in A5, and *Fusarium* was significantly enriched in A1 (Figure S3A,B).

### 3.3. Effects of Organic Fertilizer Addition on Bacterial Communities

The dominant bacterial genera detected in soils were *PSRF01*, *GWC2-73-18*, *Pseudonocardia*, *Pseudarthrobacter*__385686, *Sphingomicrobium*__483265 and *Microvirga* (Table 2). Fertilization rate exerted a significant effect on the relative abundances of *PSRF01* and *GWC2-73-18* in rhizosphere soil and *Sphingomicrobium_483265* in bulk soil (*p* < 0.05). Overall, the relative abundance of *Microvirga* peaked under treatment D1A2 in both rhizosphere and bulk soils, reaching 2.83% and 2.61%, respectively (Table 2). The highest relative abundance of *GWC2-73-18* in rhizosphere soil was 2.41% under D4A2, while the maximum value of this genus in bulk soil was 2.87% observed in treatment D1A3 (Table 2). It can be seen that organic fertilization reshaped the composition of soil bacterial communities. The relative abundance of *GWC2-73-18* increased with the rise in fertilization rate under specific mixing-ratio conditions, while *Microvirga* decreased gradually with the increase in organic fertilizer application rate. Other dominant bacterial genera remained relatively stable across different fertilization treatments.

The interaction between organic fertilizer proportion and fertilization gradient exerted an extremely significant effect on the bacterial Shannon index in bulk soil (*p* < 0.05). Compared with D3A1 and D1A1, treatment D3A3 increased the bacterial Shannon index by 1.8% in rhizosphere soil (Figure S4A), while treatment D1A5 elevated this index by 1.4% in bulk soil (Figure S4B). No significant difference was observed in Shannon index values between bulk soil and rhizosphere soil (Figure S4C,D).

Regarding fertilization rate, a total of 26 and 10 differential taxa were identified in rhizosphere soil and bulk soil, respectively (Figure S5A,B). In rhizosphere soil, nine taxa were enriched in the A5 treatment, seven taxa showed enrichment in A4 and A5, two in A3, and one in A (Figure S5A). In bulk soil, one class, *Mycobacteriales*, was enriched in A, three taxa were enriched in A3, two in A4, and three in A5 (Figure S5B).

### 3.4. Prediction of Key Microorganisms Driving Oat Yield

Random forest regression analysis was performed to identify genus-level bacterial and fungal taxa as predictive variables for oat yield. A total of three fungal genera and three bacterial genera were identified as yield predictors in rhizosphere soil (Figure 2A). Four fungal genera and six bacterial genera were screened as predictive biomarkers for yield in bulk soil, among which the genus *Pseudonocardia* from bulk soil exhibited a significant predictive effect (Figure 2B).

### 3.5. Effects of Organic Fertilizer Addition on Soil Enzyme Activities

Overall, the activities of all soil enzymes under fertilization treatments were markedly higher than that under A1 treatments. For rhizosphere soil, the maximum URE activity was observed in treatment D4A5 (Figure 3A), INV peaked at D4A4 (Figure 3C), and CAT reached the highest value under D2A5 (Figure 3G), with respective increases of 141.4%, 101.43% and 48.38% relative to D2A1. In bulk soil, the highest URE, INV and CAT activities were recorded in D3A4, D2A3 and D2A4, respectively (Figure 3B,D,H).

### 3.6. Effects of Organic Fertilizer Addition on Soil C, N and P Stoichiometric Characteristics

Overall, the maximum values of TN in either rhizosphere soil or bulk soil and SOC in bulk soil were observed under treatment D5A4, while TP peaked in D5A3; these values increased by 160.71%, 131.82% and 59.24%, 100% relative to D5A1, respectively (Table 3). For rhizosphere soil, SOC reached the highest level in D5A5 and TP in D1A4, with increments of 100.86% and 140% compared with D5A1 and D1A1. The C:N ratio of bulk soil was the highest under D1A2, and rhizosphere soil exhibited the maximum C:P ratio in D4A2 (Table 3).

### 3.7. Correlation Analysis Between Soil C, N and P Contents and Microbial Taxa

In rhizosphere soil, *Pseudogymnoascus* was positively correlated with GY, SOC, TN, URE and CAT (*p* < 0.001), INV (*p* < 0.01), and TP (*p* < 0.05). *Blastococcus* showed positive correlations with yield, SOC, URE, INV and CAT (*p* < 0.001), TP (*p* < 0.01), and TN (*p* < 0.05) (Figure 4A). In bulk soil, *Bipolaris* exhibited significantly negative correlations with yield, INV and CAT (*p* < 0.001), SOC (*p* < 0.01), and TP and URE (*p* < 0.05), whereas *GWC2-73-18* was positively correlated with CAT (*p* < 0.01), yield and SOC (*p* < 0.05) (Figure 4B).

To reveal the potential mechanism by which combined organic fertilizer application regulates oat yield, this study took organic fertilization rate as exogenous driving factors. Result from structural equation modeling indicated that organic fertilizer application rate and the soil C, N and P contents could explain 85.8% of yield (Figure 5). Organic fertilizer application rate exerted significant direct effects on yield (effect size = 0.842, *p* < 0.001) and soil C, N and P contents (effect size = 0.253, *p* < 0.05) (Figure 5). Meanwhile, organic fertilizer application rate indirectly increased the yield via positively affecting two genera (*Pseudogymnoascus* and *Blastococcus*), soil extracellular enzyme activities (URE: urease; INV: sucrase; ALP: alkaline phosphatase; CAT: catalase), and soil C, N and P contents (Figure 5).

## 4. Discussion

Yield serves as a core indicator for evaluating grassland productivity [25], and organic fertilizer application is a critical factor regulating yield increases in cultivated oat pastures. In this study, oat yield exhibited a significant increasing trend with rising organic fertilizer application rates (Figure 1), consistent with the findings of Zheng et al. [26]. Plant productivity responds variably to diverse organic fertilizer. Nevertheless, most existing studies predominantly concentrate on soil improvement driven by individual manure sources [27]. In the present study, we applied various ratios of fermented sheep manure and peat powder. The results showed that treatments with a higher proportion of fermented manure yielded slightly higher oat productivity [28]. Among all tested treatments, the regime with 20 t·ha^−1^ organic amendment and a 3:2 peat-to-fermented-sheep-manure ratio shows promise for local oat cultivation, given its maximum aboveground dry-matter yield (Figure 1). Overall, the ratio of organic fertilizer exerted a relatively minor influence on oat yield compared to the pronounced effect of total organic fertilizer application rate (Table S1). Note that changing the peat-to-fermented-sheep-manure ratio simultaneously modified inputs of organic matter, nutrients and other physicochemical constituents.

In arid and sandy marginal lands, organic fertilizer application represents a vital strategy for simultaneously achieving crop yield enhancement and soil fertility restoration, largely because organic inputs supply nutrients to soil microorganisms and facilitate the reconfiguration of microbial communities [29]. Among soil biota, fungi play an essential role in artificial grassland ecosystems [30]. Prior research indicated that most species within *Pseudogymnoascus* contribute to soil ecosystem functioning through the production of various extracellular hydrolytic enzymes that promote soil nutrient hydrolysis [31]. In this study, organic fertilizer addition significantly increased the relative abundance of *Pseudogymnoascus* in the rhizosphere (Figure 5). This pattern likely arises because organic matter addition not only provided essential nutrients and energy for its growth but also improved soil aeration and moisture conditions via co-amendment with fermented sheep manure and peat [32], thereby creating favorable microhabitats for proliferation. In addition, genera including *Fusarium*, *Alternaria*, and *Bipolaris* were detected in our soil samples. The relative abundances of *Alternaria* and *Bipolaris* declined with increasing organic-amendment application (Table 1). Many studies have reported that some species within these genera contain phytopathogenic members that trigger crop diseases and economic losses [33,34]. Although genus-level taxonomic resolution cannot confirm the pathogenic potential of the specific taxa observed here, the reduced relative abundances of *Alternaria* and *Bipolaris* under organic amendment may be beneficial for crop yield and soil environment.

Furthermore, bacteria represent the most abundant and taxonomically diverse microbial group on Earth, governing critical ecological processes such as soil nutrient turnover and energy flow [35]. Studies have demonstrated that nutrient enrichment significantly increases soil bacterial richness and abundance [36]. Genera such as *Blastococcus* and *GWC2-73-18* [37] perform multiple functions in soil nutrient cycling. *Blastococcus* species have been found among the keystone taxa in soils affected by various challenging conditions, such as salinity, alkalinity, toxic amendments [38], and, notably, heavy metal contamination [39,40,41]. The strains also exhibited plant growth-promoting traits, enhanced heavy metal resistance, and the ability to degrade environmental pollutants, positioning *Blastococcus* as a candidate for sustainable agriculture and bioremediation [42]. In this study, the response of *Blastococcus* and *GWC2-73-18* to fertilization rates indicated that high nutrient concentrations were accompanied by elevated relative abundances of these two genera (Table 2). Meanwhile, correlation analyses between yield-predictive microbial genera and soil properties revealed that only rhizosphere *Pseudogymnoascus* and *Blastococcus* exhibited significantly positive correlations with soil enzyme activities, soil nutrients, and yield (Figure 4). Structural equation modeling further confirmed that increased fertilization was directly associated with higher relative abundances of both *Pseudogymnoascus* and *Blastococcus* (Figure 5). Given the recognized beneficial roles of these two genera, our findings suggest that organic fertilizer application contributes to optimizing soil microbial community composition.

Microorganisms enriched in soil secrete a diverse array of extracellular enzymes, among which certain enzymes participate in the decomposition and synthesis of soil organic matter as well as in the cycling of carbon (C), nitrogen (N), and phosphorus (P) [43,44]. Soil invertase (sucrase) hydrolyzes sucrose into monosaccharides (glucose and fructose), serving as a key catalyst in soil C cycling [38]. Urease decomposes organic N into ammonia and carbon dioxide, rendering N directly available to plants [45]. Soil phosphatase specifically hydrolyzes organic P compounds into inorganic phosphate, facilitating plant P uptake and constituting a rate-limiting enzyme in soil P cycling [46]. Organic fertilizers can modulate soil enzyme activities through alterations in microbial community structure [47,48,49]. In the present study, the activities of soil invertase and urease, as well as soil C and N contents, increased significantly with rising fertilization rates (Figure 3A–D, Table 3). This indicates that organic matter addition provided adequate nutrients for soil microorganisms, influenced bacterial and fungal community composition, promoted the secretion of key enzymes, and ultimately enhanced soil C and N pools. Xie et al. [50] reported that organic fertilizer application significantly increased soil phosphatase activity. However, in our study, organic amendment did not significantly affect phosphatase activity, despite a marked increase in soil total P content. This discrepancy may be attributable to the fact that organic P in the fermented manure had been largely mineralized prior to application, thereby increasing soil P availability while few residual organic P substrates remained to stimulate phosphatase activity. In addition to the enzymes involved in C, N, and P cycling, catalase plays a crucial role in decomposing hydrogen peroxide, thereby protecting soil from oxidative stress, particularly in the rhizosphere. In this study, catalase activity increased with rising organic fertilizer application rates, likely because organic fertilizer altered bacterial species composition and selectively recruited bacteria that promote catalase activity, corroborating the findings of Fang et al. [51]. Notably, the enrichment of *Pseudogymnoascus* and *Blastococcus* exerted significant positive effects on the activities of the aforementioned enzymes. This indicates that the composition of microbial communities is significantly correlated with organic fertilizer application, soil enzyme activities, and the availability of soil carbon, nitrogen and phosphorus(Figure 6). Importantly, the results obtained from the Horqin Sandy Land in this study are regionally representative. For typical transitional ecosystems characterized by alternating wet and dry climates and agro-pastoral production, our findings can serve as a demonstration case for desertified grasslands in northern China and provide field-based theoretical evidence and technical references.

This study was conducted at a single site and in a single growing season, which limits the generalizability of our results. Variations in rainfall, temperature, peat and manure properties, previous land use, and inherent soil conditions may further alter fertilization performance under field conditions. Therefore, these findings provide reliable references for local oat cultivation under similar field conditions, and further multi-site and multi-year investigations will help extend and generalize the current conclusions. Importantly, peat is regarded as a strategic mineral resource for emerging industries with an extremely long formation cycle and poor renewability. Large-scale peat extraction destroys peatland wetland ecosystems and releases long-term sequestered soil carbon, exacerbating terrestrial carbon loss and restricting the sustainable agricultural application of peat materials. In this context, developing local carbon-rich alternatives, such as biochar, straw compost, and mushroom residue, has become a crucial research direction for sustainable sandy soil improvement. In addition, due to the absence of nutrient-equivalent mineral control, we cannot fully disentangle whether plant and soil responses stem from extra nutrient supply, organic-matter inputs, or other intrinsic properties of organic fertilizer. Our results thus reflect comprehensive effects of combined organic fertilizer rather than isolated nutrient effects. Nevertheless, under sandy soil conditions with low fertility and poor structural stability, numerous studies have confirmed the unique soil-improving advantages of organic fertilizers over single mineral fertilization. The primary objective of our study was to screen suitable application ratios and rates of compound organic fertilizer for field production optimization, rather than to independently verify nutrient-related mechanisms. In this practical-oriented context, our current experimental design is valid and sufficient to support the conclusions of this study.

## 5. Conclusions

In desert lands of northern China, this field-based experiment showed that organic fertilizer application confers dual benefits of increasing crop yield and improving soil quality. Overall, increasing organic fertilizer application rates within a reasonable range significantly promoted oat dry-matter yield, whereas excessive fertilization restricted further yield improvement and led to yield reduction under the D5 treatment. The ratio of peat powder to fermented sheep manure exerted a relatively weak regulatory effect on oat yield, and the optimal ratio varied across different fertilization rates. Among all treatments, the combination with an application rate of 20 t·ha^−1^ and a peat-powder-to-fermented-sheep-manure ratio of 3:2 achieved the maximum oat yield, representing the optimal fertilization regime for local oat cultivation in sandy lands. Organic fertilizers increased the relative abundances of Pseudogymnoascus and Blastococcus, optimizing soil microbial community composition, and these two genera showed significant correlations with soil enzyme activities. Urease, invertase, and catalase exhibited significant effects on soil organic carbon, total nitrogen, and total phosphorus contents, whereas alkaline phosphatase predominantly participated in the transformation of soil organic carbon and total phosphorus. The collective elevation of enzyme activities enhanced microbial mineralization efficiency, thereby improving soil nutrient pools and nutrient supply capacity. Ultimately, these improvements in the soil nutrient environment for oat growth translated into substantial increases in oat yield. Furthermore, the Horqin region represents a typical semi-arid agro-pastoral ecotone, and the findings of this study provide important references for semi-arid regions in northern China.

## Figures and Tables

**Figure 1:**
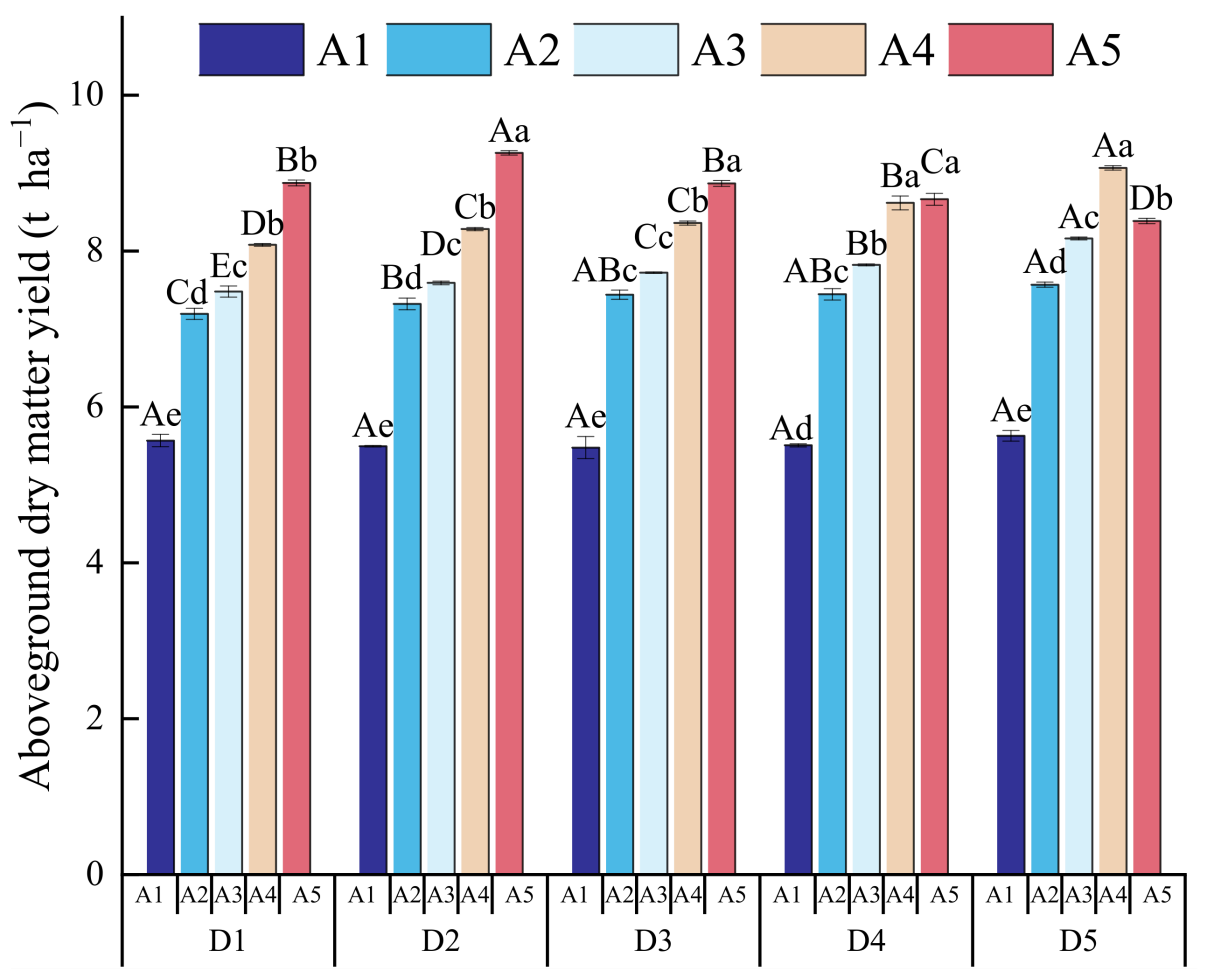
Interaction of organic fertilizer proportion and fertilization rate on oat yield. A represents five application rates (0, 5, 10, 15, and 20, t·ha^−1^; A1–A5), and D represents five ratios (peat powder:fermented manure: 7:3, 3:2, 1:1, 2:3, 3:7; D1–D5). Values represent means ± SE. (*n* = 4). Capital letters indicate significant differences among organic fertilizer proportions under the same fertilization rate, while lowercase letters denote significant differences among fertilization rates with the same organic fertilizer proportion.

**Figure 2:**
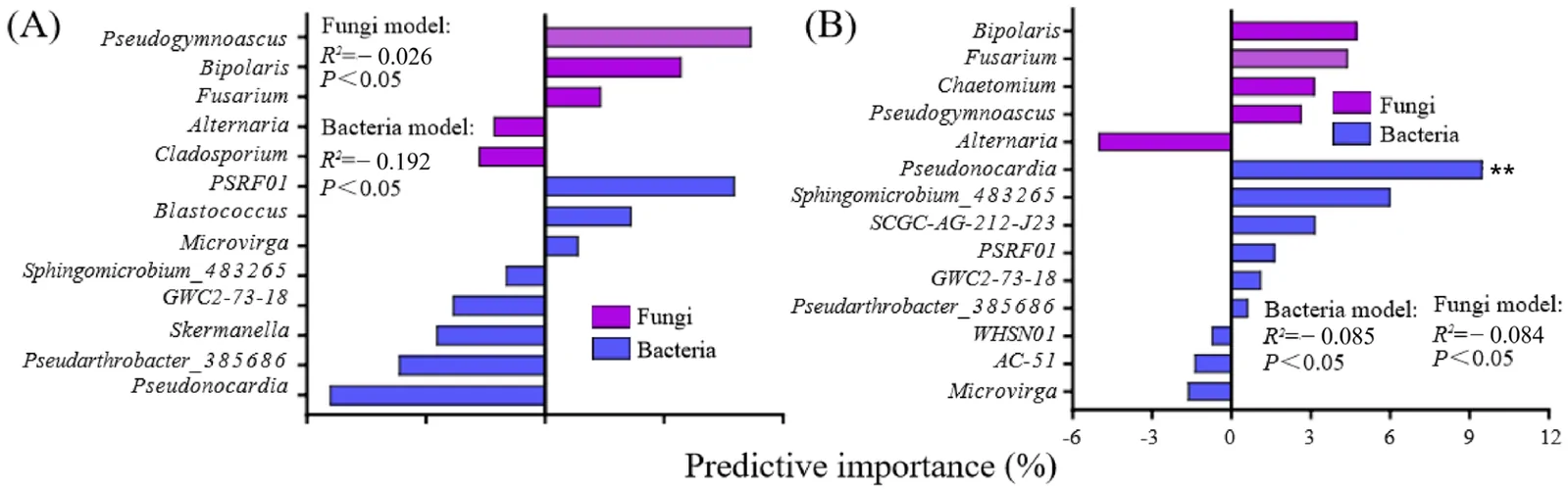
Random forest regression for exploring potential associations between oat yield and bacterial and fungal genus-level taxa. (**A**) represents rhizosphere soil; (**B**) represents bulk soil. This analysis was only used to screen microbial genera with explanatory associations with oat yield and was not an out-of-sample predictive model. **, *p* < 0.01, indicates that the variable importance of the microbial genus reached an extremely significant level based on permutation test.

**Figure 3:**
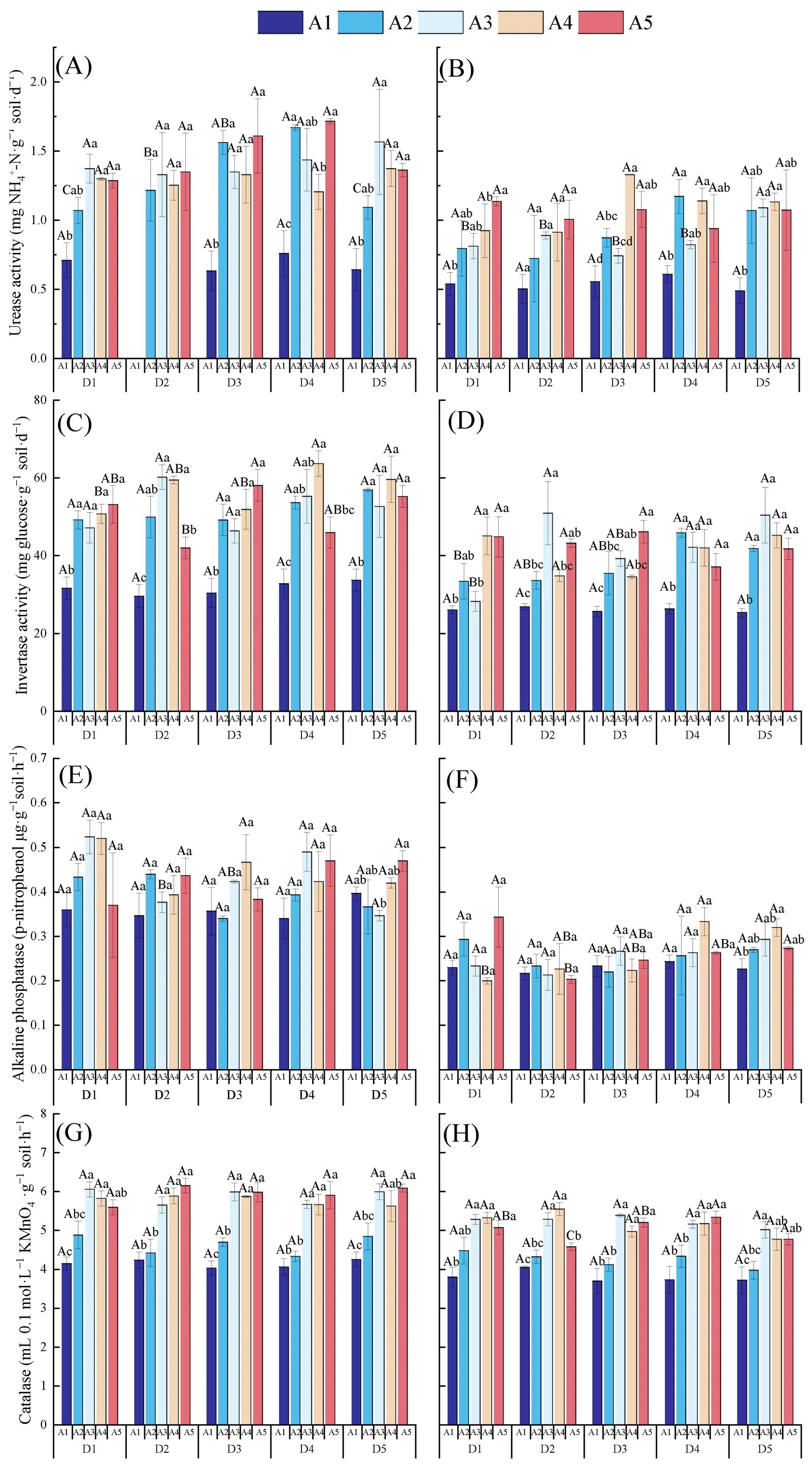
Effects of different organic fertilizer proportions and application rates on soil enzyme activities. (**A**,**B**) Urease activity; (**C**,**D**) invertase activity; (**E**,**F**) alkaline phosphatase activity; (**G**,**H**) catalase activity. Panels (**A**,**C**,**E**,**G**) represent rhizosphere soil, while panels (**B**,**D**,**F**,**H**) represent bulk soil. Values represent means ± SE (*n* = 4).

**Figure 4:**
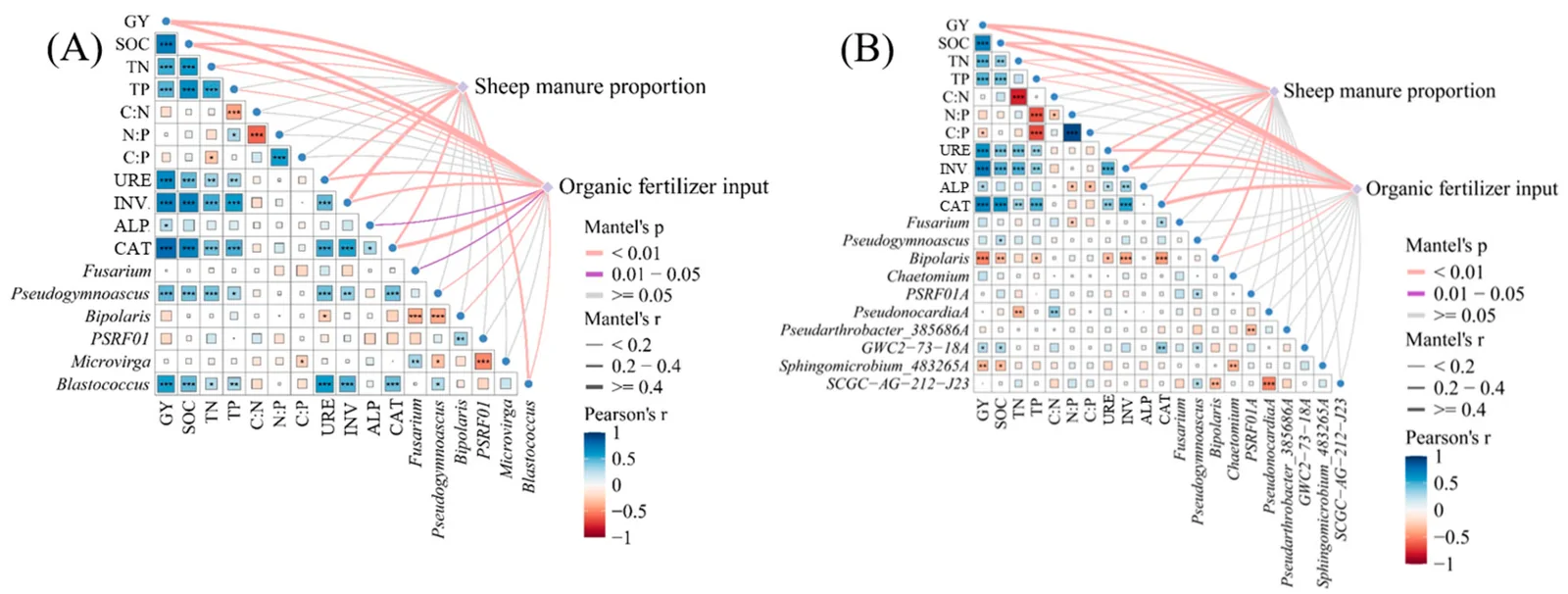
Correlations of fertilization with microbial genera, soil enzyme activities, soil nutrients and oat yield. (**A**) rhizosphere soil; (**B**) bulk soil. *, *p* < 0.05, indicates significant difference; **, *p* < 0.01, indicates extremely significant difference; ***, *p* < 0.001, indicates highly extremely significant difference based on permutation test.

**Figure 5:**
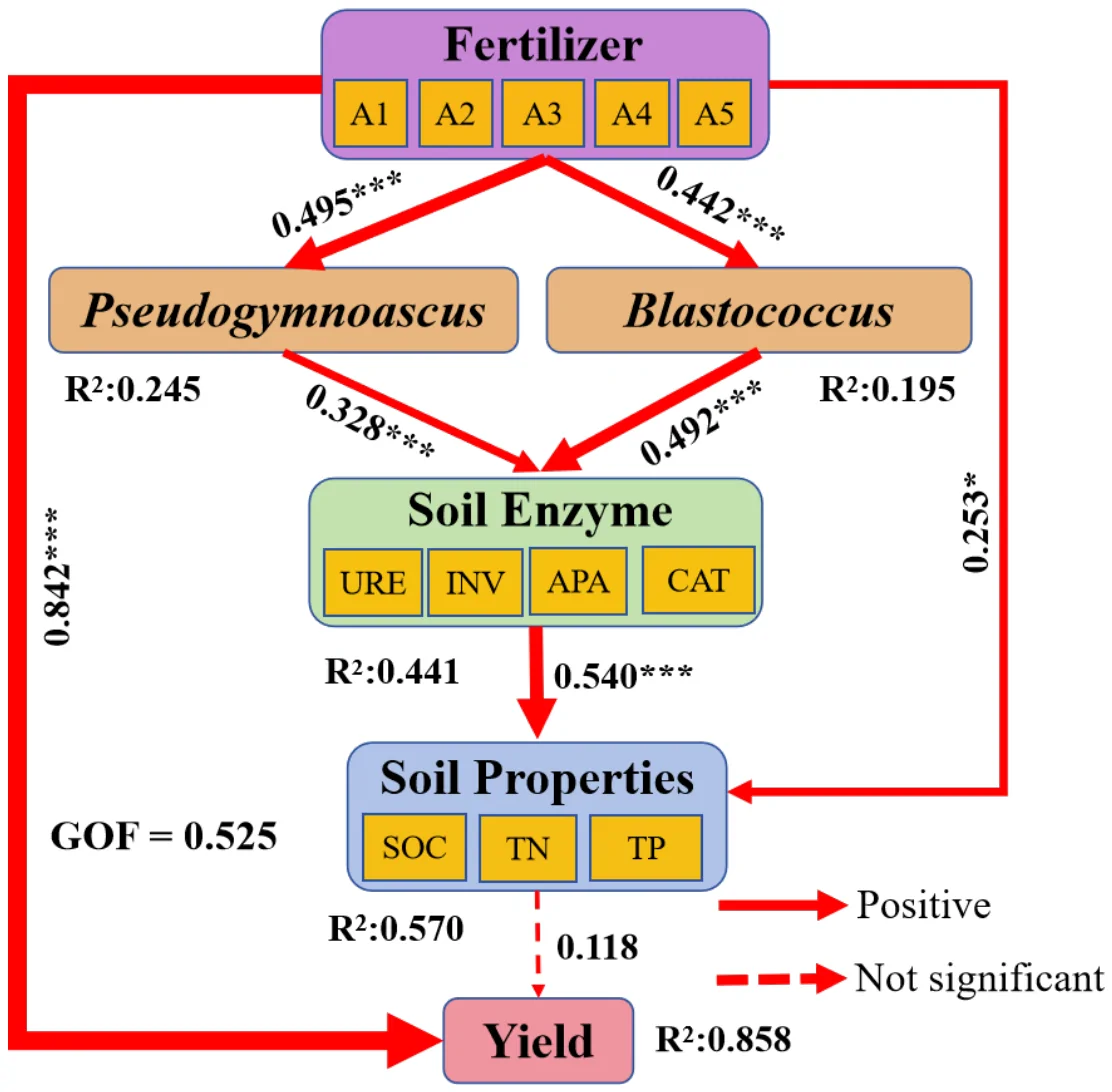
Structural equation model exploring correlational associations among microorganisms, soil enzyme activities, soil nutrients and oat yield. * and *** indicate a significant path at *p* < 0.05 and *p* < 0.001, respectively. Dashed line, non-significant differences; solid line, positive path. The thickness of lines indicates the contribution size. *n* = 100. Model acceptance criteria: CFI and TLI > 0.90, RMSEA ≤ 0.08, and SRMR ≤ 0.08.

**Figure 6:**
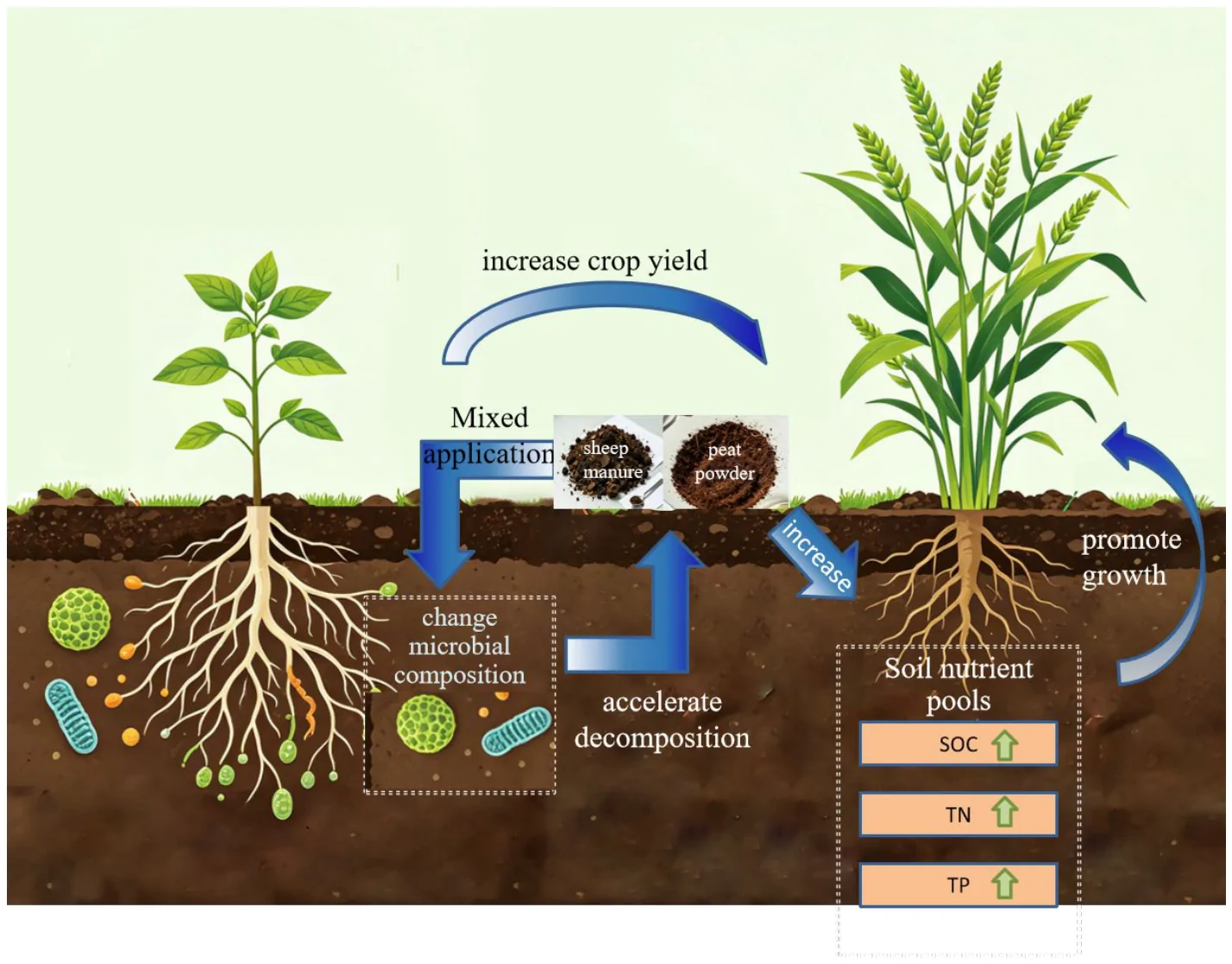
Mechanisms underlying the effects of organic fertilizer amendment on oat yield. Co-application of manure and peat powder reshaped microbial community assemblages, boosted the decomposition of exogenous organic fertilizer, and raised soil carbon, nitrogen and phosphorus pools. These nutrients further promoted vegetative and reproductive growth of oats, which ultimately contributed to higher aboveground dry-matter yield.

**Table 1:** Effects of organic fertilizer proportions on fungal community composition.

Genus		*Fusarium* (%)		*Alternaria* (%)		*Pseudogymnoascus* (%)		*Bipolaris* (%)	
		Rhizosphere Soil	Bulk Soil	Rhizosphere Soil	Bulk Soil	Rhizosphere Soil	Bulk Soil	Rhizosphere Soil	Bulk Soil
D1	A1	10.33 ± 2.13 Aa	8.22 ± 0.92 Ab	15.85 ± 2.36 Aab	6.37 ± 2.43 Aa	2.32 ± 0.5 Aa	6.03 ± 1.19 Aa	15.07 ± 1.54 Aa	4.24 ± 2.37 Aa
D1	A2	8.59 ± 1.89 Ba	14.76 ± 0.78 Aa	19.52 ± 0.69 Aa	0.88 ± 0.51 Ba	2.7 ± 0.58 Aa	3 ± 1.22 Aa	19.07 ± 2.45 ABa	1.29 ± 0.41 Aa
D1	A3	12.58 ± 2.48 Aa	13.98 ± 1.57 Aa	16.77 ± 2.38 Aab	3.13 ± 1.12 Aa	3.63 ± 0.97 Aa	9.04 ± 0.93 Aa	12.41 ± 0.43 Aa	2.37 ± 0.69 Aa
D1	A4	10.56 ± 2.52 Aa	12.82 ± 1.06 Aab	13.55 ± 2.18 Aab	1.62 ± 0.6 Aa	2.67 ± 0.44 Aa	6.24 ± 3.05 Aa	17.66 ± 2.46 Aa	1.37 ± 0.22 Ba
D1	A5	10.08 ± 2.64 Aa	12.22 ± 2.81 Aab	10.32 ± 0.87 Bb	2.95 ± 1.34 ABa	3.65 ± 1.34 ABa	5.61 ± 2.52 Aa	13.24 ± 6.98 Aa	1.63 ± 0.65 Aa
D2	A1	11.56 ± 2.29 Aab	7.50 ± 0.46 Aa	16.12 ± 3.79 Aa	10.43 ± 1.1 Aa	1.86 ± 0.64 Ab	5.78 ± 1.92 Aa	16.34 ± 1.23 Aa	7.45 ± 2.56 Aa
D2	A2	8.45 ± 0.55 Bb	11.12 ± 2.73 Aa	22.93 ± 4.02 Aa	2.2 ± 0.57 ABb	1.97 ± 0.17 Ab	5.59 ± 3.26 Aa	16.18 ± 1.04 ABa	1.91 ± 0.87 Aa
D2	A3	9.33 ± 0.98 Ab	12.46 ± 0.91 ABa	16.25 ± 2.79 Aa	1.73 ± 0.25 Ab	3.54 ± 0.35 Aa	7.78 ± 1.00 Aa	12.52 ± 4.72 Aa	1.63 ± 0.6 Aa
D2	A4	9.03 ± 0.62 Ab	20.88 ± 8.46 Aa	16.57 ± 3.00 Aa	3.84 ± 0.94 Aab	3.64 ± 0.27 Aa	9.08 ± 0.2 Aa	13.34 ± 2.59 Aa	2.46 ± 0.36 Aa
D2	A5	14.75 ± 2.10 Aa	13.18 ± 2.23 Aa	14.69 ± 1.34 ABa	1.12 ± 0.07 Bb	3.64 ± 0.53 ABa	5.8 ± 0.24 Aa	11.44 ± 2.13 Aa	0.73 ± 0.1 Aa
D3	A1	8.84 ± 1.87 Aa	8.25 ± 1.13 Ab	17.54 ± 2.01 Aa	5.54 ± 2.8 Aa	1.83 ± 0.16 Ab	5.02 ± 0.81 Aa	16.34 ± 1.07 Aa	1.96 ± 0.81 Aa
D3	A2	14.69 ± 2.15 Aa	11.78 ± 1.41 Aab	14.60 ± 1.25 Aa	3.98 ± 1.03 Aab	2.94 ± 0.54 Ab	3.16 ± 0.82 Aa	9.9 ± 1.5 Cbc	1.45 ± 0.21 Aa
D3	A3	7.24 ± 1.40 Aa	13.79 ± 1.08 Aa	13.85 ± 1.69 Aa	2.9 ± 1.02 Aab	2.72 ± 0.63 Ab	4.97 ± 0.41 Aa	16.88 ± 1.92 Aa	1.33 ± 0.38 Aa
D3	A4	12.74 ± 1.79 Aa	11.53 ± 1.37 Aab	15.63 ± 1.01 Aa	2.99 ± 0.67 Aab	3.06 ± 0.84 Ab	3.4 ± 1.14 Aa	13.75 ± 1.82 Aab	1.32 ± 0.25 Ba
D3	A5	15.16 ± 5.65 Aa	10.03 ± 1.76 Aab	15.69 ± 3.4 ABa	1.63 ± 0.39 Bb	6.46 ± 0.97 Aa	3.83 ± 1 Aa	8.54 ± 1.09 Ac	1.5 ± 0.32 Aa
D4	A1	9.10 ± 2.62 Aa	8.95 ± 1.35 Ab	15.57 ± 1.51 Aa	2.31 ± 0.06 Aa	1.77 ± 0.22 Aa	5.28 ± 0.71 Aa	17.57 ± 0.11 Aa	1.03 ± 0.12 Aa
D4	A2	9.56 ± 1.46 Ba	12.33 ± 0.88 Aa	15.75 ± 2.48 Aa	2.46 ± 0.59 ABa	2.81 ± 1.2 Aa	5.1 ± 1.63 Aa	14.48 ± 2.3 BCa	1.4 ± 0.35 Aa
D4	A3	8.65 ± 1.49 Aa	13.44 ± 0.92 Aa	15.06 ± 1.6 Aa	2.39 ± 1.00 Aa	2.02 ± 0.61 Aa	6.15 ± 3.55 Aa	15.21 ± 1.23 Aa	1.47 ± 0.54 Aa
D4	A4	7.98 ± 2.81 Aa	8.47 ± 0.16 Ab	15.75 ± 0.8 Aa	2.79 ± 1.00 Aa	2.36 ± 0.51 Aa	6.68 ± 3.12 Aa	18.03 ± 5.72 Aa	0.65 ± 0.1 Ba
D4	A5	11.39 ± 2.69 Aa	11.92 ± 1.4 Aa	17.99 ± 1.14 Aa	4.54 ± 1.35 ABa	2.95 ± 0.4 Ba	5.69 ± 1.75 Aa	10.7 ± 1.14 Aa	1.96 ± 0.6 Aa
D5	A1	9.51 ± 0.07 Aa	7.34 ± 0.35 Aa	16.65 ± 2.72 Aa	7.7 ± 2.49 Aa	1.60 ± 0.5 Aa	6.29 ± 1.42 Aa	14.19 ± 1.55 Aa	5.37 ± 2.56 Aa
D5	A2	8.51 ± 0.83 Ba	13.38 ± 3.91 Aa	16.31 ± 2.35 Aa	2.94 ± 0.54 ABa	2.24 ± 0.7 Aa	7.13 ± 0.98 Aa	21.58 ± 1.17 Aa	1.67 ± 0.58 Aa
D5	A3	7.46 ± 2.74 Aa	9.36 ± 0.71 Ba	14.52 ± 1.12 Aa	2.82 ± 1.23 Aa	2.41 ± 0.59 Aa	6.39 ± 1.9 Aa	15.92 ± 1.98 Aa	2.02 ± 0.75 Aa
D5	A4	9.77 ± 0.49 Aa	9.19 ± 0.64 Aa	11.18 ± 2 Aa	5.52 ± 3.37 Aa	2.82 ± 0.36 Aa	10.21 ± 4.12 Aa	17.07 ± 5.34 Aa	1.44 ± 0.45 Ba
D5	A5	18.67 ± 8.42 Aa	9.34 ± 0.45 Aa	14.06 ± 1.98 ABa	5.82 ± 1.43 Aa	2.93 ± 0.35 Ba	8.99 ± 2.69 Aa	12.49 ± 1.86 Aa	3.7 ± 1.74 Aa

For all measured variables, A represents fertilization application rate and D represents five ratios (peat powder:fermented manure: 7:3, 3:2, 1:1, 2:3, 3:7; D1–D5). Values represent means ± SE (*n* = 4). Capital letters mark significant differences among various organic fertilizer proportions under an identical fertilization rate, whereas lowercase letters denote significant differences across fertilization rates for the same organic fertilizer proportion. This labeling criterion applies to all subsequent tables and figures. The genera with total relative abundance > 1% across all samples were retained.

**Table 2:** Effects of organic fertilizer proportions and fertilization rates on bacterial community composition.

Genus		*PSRF01* (%)		*Pseudonocardia* (%)		*Microvirga* (%)		*Pseudarthrobacter*_385686 (%)		*GWC2-73-18* (%)		***Sphingomicrobium*** **_483265 (%)**	***Blastococcus***
		Rhizosphere Soil	Bulk Soil	Rhizosphere Soil	Bulk Soil	Rhizosphere Soil	Bulk Soil	Rhizosphere Soil	Bulk Soil	Rhizosphere Soil	Bulk Soil	**Rhizosphere Soil**	**Rhizosphere Soil**
D1	A1	3.58 ± 0.35 Aa	4.39 ± 0.16 Aa	2.27 ± 0.55 Aa	1.72 ± 0.33 Aab	2.22 ± 0.27 Aab	1.10 ± 0.16 Aa	2.03 ± 0.11 Aab	1.57 ± 0.17 Aa	1.68 ± 0.25 Aa	1.98 ± 0.33 Aa	1.29 ± 0.15 Aa	1.16 ± 0.11 Aa
	A2	2.69 ± 0.34 Aa	4.81 ± 0.84 Aa	2.83 ± 0.35 Aa	2.38 ± 0.35 Aa	2.61 ± 0.25 Aa	1.48 ± 0.34 Aa	2.11 ± 0.28 Aab	1.24 ± 0.09 Aa	1.76 ± 0.08 Aa	2.43 ± 0.29 Aa	1.46 ± 0.06 Aa	1.34 ± 0.77 Aab
	A3	2.94 ± 0.06 Aa	4.83 ± 0.71 Aa	2.25 ± 0.2 Aa	1.34 ± 0.66 Aab	2.12 ± 0.21 Aab	1.06 ± 0.31 Aa	2.11 ± 0.19 Aab	1.08 ± 0.33 Aa	1.87 ± 0.41 Aa	2.87 ± 0.58 Aa	1.44 ± 0.05 Aa	1.13 ± 0.65 Ab
	A4	3.06 ± 0.52 Aa	3.37 ± 0.2 Aa	2.23 ± 0.23 Aa	0.97 ± 0.06 Ab	2.18 ± 0.34 Aab	0.97 ± 0.03 Aa	2.42 ± 0.22 Aa	1.59 ± 0.44 Aa	2.1 ± 0.16 Aa	1.68 ± 0.08 Ba	1.24 ± 0.08 Aa	1.60 ± 0.93 Aa
	A5	3.21 ± 0.25 Aa	3.76 ± 0.09 Ba	2.34 ± 0.08 Aa	1.51 ± 0.09 Aab	1.61 ± 0.12 Bb	1.24 ± 0.07 Aa	1.61 ± 0.14 Bb	1.23 ± 0.14 Aa	2.13 ± 0.22 Aa	1.7 ± 0.3 Ba	1.19 ± 0.08 Aa	1.35 ± 0.78 Aab
D2	A1	3.17 ± 0.2 Aa	4.38 ± 0.21 Aa	2.16 ± 0.31 Aa	2.24 ± 0.21 Aa	2.6 ± 0.27 Aa	1.37 ± 0.06 Aa	2.03 ± 0.19 Aa	1.34 ± 0.08 Aa	1.59 ± 0.41 Aa	1.58 ± 0.07 Aa	1.44 ± 0.18 Aa	1.20 ± 0.14 Aa
	A2	2.73 ± 0.56 Aa	4.41 ± 0.13 Aa	2.6 ± 0.23 ABa	1.57 ± 0.2 Aa	2.42 ± 0.24 Aa	1.35 ± 0.26 Aa	1.72 ± 0.29 Aa	1.29 ± 0.08 Aa	1.92 ± 0.11 Aa	1.83 ± 0.5 Aa	1.29 ± 0.1 Aa	1.44 ± 0.14 Aa
	A3	3.46 ± 0.53 Aa	4.64 ± 0.48 Aa	2.64 ± 0.34 Aa	1.71 ± 0.32 Aa	2.3 ± 0.17 Aa	1.06 ± 0.04 Aa	2.02 ± 0.1 Aa	1.5 ± 0.18 Aa	2.35 ± 0.38 Aa	2.21 ± 0.25 ABa	1.25 ± 0.18 Aa	1.39 ± 0.05 Aa
	A4	2.91 ± 0.14 Aa	6.03 ± 0.97 Aa	2.45 ± 0.33 Aa	1.37 ± 0.06 Aa	2.08 ± 0.19 Aa	0.95 ± 0.14 Aa	2.22 ± 0.46 Aa	1.17 ± 0.06 Aa	1.91 ± 0.11 Aa	2.69 ± 0.32 Aa	1.31 ± 0.15 Aa	1.37 ± 0.13 Aa
	A5	2.05 ± 0.1 Ba	4.41 ± 1.09 ABa	2.04 ± 0.11 Aa	2.21 ± 0.98 Aa	2.23 ± 0.25 ABa	1.21 ± 0.27 Aa	2.44 ± 0.12 Aa	1.35 ± 0.12 Aa	1.76 ± 0.39 Aa	2.44 ± 0.19 ABa	1.53 ± 0.03 Aa	1.35 ± 0.06 Aa
D3	A1	3.61 ± 0.43 Aa	4.29 ± 0.15 Aa	1.82 ± 0.39 Aa	1.65 ± 0.39 Aa	2.22 ± 0.33 Aa	0.99 ± 0.14 Aa	2.12 ± 0.09 Aa	1.69 ± 0.15 Aa	1.52 ± 0.23 Ab	2.09 ± 0.39 Aa	1.33 ± 0.18 Aa	1.21 ± 0.13 Aa
	A2	3.71 ± 0.33 Aa	3.05 ± 0.58 Aa	1.77 ± 0.22 Ba	1.66 ± 0.25 Aa	2.02 ± 0.29 Aa	1.45 ± 0.21 Aa	2.24 ± 0.18 Aa	1.66 ± 0.15 Aa	1.87 ± 0.06 Aab	1.88 ± 0.13 Aa	1.28 ± 0.13 Aa	1.11 ± 0.12 Aa
	A3	3.32 ± 0.34 Aa	3.96 ± 0.24 Aa	2.42 ± 0.19 Aa	1.37 ± 0.08 Aa	1.79 ± 0.11 Aa	1.15 ± 0.08 Aa	1.77 ± 0.32 Aa	1.26 ± 0.08 Aa	2.12 ± 0.19 Aab	1.84 ± 0.24 ABa	1.3 ± 0.13 Aa	1.33 ± 0.20 Aa
	A4	2.95 ± 0.42 Aa	5.53 ± 2.4 Aa	2.39 ± 0.3 Aa	1.5 ± 0.63 Aa	2.25 ± 0.39 Aa	1.18 ± 0.19 Aa	1.93 ± 0.04 Aa	1.67 ± 0.44 Aa	1.93 ± 0.29 Aab	2.38 ± 0.35 ABa	1.47 ± 0.1 Aa	1.44 ± 0.08 Aa
	A5	3.14 ± 0.29 Aa	3.91 ± 0.19 ABa	2.28 ± 0.23 Aa	1.37 ± 0.19 Aa	1.87 ± 0.34 ABa	0.94 ± 0.1 Aa	2.16 ± 0.21 ABa	1.49 ± 0.24 Aa	2.35 ± 0.12 Aa	1.76 ± 0.08 Ba	1.24 ± 0.18 Aa	1.56 ± 0.11 Aa
D4	A1	3.98 ± 0.41 Aa	4.40 ± 0.18 Aa	1.37 ± 0.08 Aa	1.21 ± 0.14 Aa	1.83 ± 0.03 Aa	0.84 ± 0.08 Aa	2.02 ± 0.04 Aa	1.81 ± 0.14 Aa	1.78 ± 0.08 Aa	1.39 ± 0.4 Ab	1.14 ± 0.10 Aa	1.16 ± 0.15 Aa
	A2	3.48 ± 0.67 Aa	3.93 ± 0.34 Aa	2.32 ± 0.3 ABa	1.56 ± 0.14 Aa	1.98 ± 0.51 Aa	1.1 ± 0.07 Aa	2.29 ± 0.48 Aa	1.53 ± 0.28 Aa	2.41 ± 0.69 Aa	2.16 ± 0.42 Aab	1.14 ± 0.11 Aa	1.18 ± 0.13 Aa
	A3	3.32 ± 0.22 Aa	3.34 ± 0.22 Aa	2.56 ± 0.44 Aa	1.49 ± 0.22 Aa	2.01 ± 0.19 Aa	1.19 ± 0.32 Aa	1.55 ± 0.21 Aa	1.08 ± 0.14 Aa	2.2 ± 0.12 Aa	1.36 ± 0.06 Bb	1.33 ± 0.11 Aa	1.38 ± 0.03 Aa
	A4	2.82 ± 0.51 Aa	3.43 ± 1.04 Aa	2.24 ± 0.32 Aa	1.29 ± 0.08 Aa	1.78 ± 0.16 Aa	0.92 ± 0.11 Aa	1.65 ± 0.34 Aa	1.12 ± 0.06 Aa	1.63 ± 0.2 Aa	1.43 ± 0.28 Bb	1.21 ± 0.18 Aa	1.37 ± 0.21 Aa
	A5	2.94 ± 0.28 Aa	4.24 ± 0.06 ABa	2.13 ± 0.22 Aa	1.34 ± 0.25 Aa	1.88 ± 0.08 ABa	0.85 ± 0.09 Aa	2.16 ± 0.25 ABa	1.35 ± 0.05 Aa	2.13 ± 0.24 Aa	2.66 ± 0.16 Aa	1.3 ± 0.16 Aa	1.32 ± 0.13 Aa
D5	A1	3.63 ± 0.43 Aa	4.30 ± 0.16 Aa	2.60 ± 0.53 Aa	1.96 ± 0.28 Aa	2.36 ± 0.28 Aa	1.23 ± 0.12 Aa	2.05 ± 0.13 Aa	1.56 ± 0.20 Aa	1.69 ± 0.31 Ab	1.66 ± 0.09 Aa	1.39 ± 0.14 Aa	1.06 ± 0.05 Ab
	A2	4.07 ± 0.85 Aa	3.46 ± 0.44 Aa	2.44 ± 0.32 ABa	1.61 ± 0.21 Aa	1.87 ± 0.41 Aa	1.37 ± 0.26 Aa	1.69 ± 0.52 Aa	1.51 ± 0.15 Aa	2.18 ± 0.1 Aab	2.28 ± 0.26 Aa	1.46 ± 0.21 Aa	1.45 ± 0.15 Aa
	A3	3.08 ± 0.4 Aa	4.71 ± 0.66 Aa	2.02 ± 0.26 Aa	1.49 ± 0.07 Aa	2 ± 0.25 Aa	1.31 ± 0.18 Aa	1.83 ± 0.18 Aa	1.54 ± 0.26 Aa	2.37 ± 0.08 Aa	1.84 ± 0.19 ABa	1.28 ± 0.18 Aa	1.49 ± 0.13 Aa
	A4	3.25 ± 0.13 Aa	4.51 ± 0.94 Aa	2.37 ± 0.64 Aa	1.12 ± 0.09 Aa	2.13 ± 0.11 Aa	0.99 ± 0.15 Aa	1.71 ± 0.2 Aa	1.19 ± 0.26 Aa	2.18 ± 0.16 Aab	1.95 ± 0.34 ABa	1.13 ± 0.1 Aa	1.43 ± 0.10 Aa
	A5	2.67 ± 0.09 ABa	5.14 ± 0.06 Aa	2.54 ± 0.29 Aa	1.95 ± 0.38 Aa	2.41 ± 0.21 Aa	1.19 ± 0.13 Aa	2.07 ± 0.11 ABa	1.27 ± 0.06 Aa	1.63 ± 0.14 Ab	2.43 ± 0.36 ABa	1.5 ± 0.08 Aa	1.14 ± 0.1 Aab

Note: For all measured variables, A represents fertilization application rate and D represents organic fertilizer mixing proportion. Values represent means ± SE (*n* = 4). Capital letters mark significant differences among various organic fertilizer proportions under an identical fertilization rate, whereas lowercase letters denote significant differences across fertilization rates for the same organic fertilizer proportion. This labeling criterion applies to all subsequent tables and figures. The genera with total relative abundance > 1% across all samples were retained.

**Table 3:** Effects of different organic fertilizer proportions on soil C:N:P stoichiometry.

		SOC (g/kg)		TN (g/kg)		TP (g/kg)		C:N		N:P		**C** **:** **P**	
D	A	Rhizosphere Soil	Bulk Soil	Rhizosphere Soil	Bulk Soil	Rhizosphere Soil	Bulk Soil	Rhizosphere Soil	Bulk Soil	Rhizosphere Soil	Bulk Soil	**Rhizosphere Soil**	**Bulk Soil**
D1	A1	3.43 ± 0.26 Ab	3.62 ± 0.15 Ac	0.34 ± 0.19 Aa	0.33 ± 0.05 Aab	0.03 ± 0.01 Bb	0.06 ± 0.02 Aa	15.26 ± 13.5 Aa	11.12 ± 2.18 Ab	10.2 ± 6.19 Aa	5.85 ± 0.93 Ba	107.34 ± 30.32 Aa	65.24 ± 16.41 Ba
	A2	4.85 ± 1.05 Aab	4.3 ± 0.16 Ab	0.58 ± 0.16 Aa	0.19 ± 0.04 Bb	0.06 ± 0.02 Abc	0.06 ± 0.02 ABa	8.67 ± 1.77 Ba	22.69 ± 3.7 Aa	9.92 ± 4.11 Aa	3.12 ± 0.28 Ab	81.45 ± 16.13 Aa	70.91 ± 14.21 Aa
	A3	4.68 ± 1.03 Aab	4.9 ± 0.11 ABab	0.7 ± 0.43 Aa	0.32 ± 0.07 Aab	0.07 ± 0.02 Ab	0.08 ± 0.01 BCa	8.49 ± 4.47 Ba	15.79 ± 3.05 Ab	11.24 ± 8.19 Aa	4.03 ± 0.92 Bab	71.46 ± 1.13 Aab	62.25 ± 9.53 Ba
	A4	5.03 ± 0.2 Aab	5.27 ± 0.11 Aa	0.6 ± 0.2 ABa	0.49 ± 0.15 Aa	0.12 ± 0.02 Ab	0.09 ± 0.02 Aa	9.02 ± 3.07 Ba	11.35 ± 3.2 Bb	5.1 ± 2.25 Aa	5.32 ± 0.56 Aab	41.44 ± 7.15 Bb	59.25 ± 11.41 Aa
	A5	6.18 ± 1.52 Aa	4.74 ± 0.72 Aab	0.5 ± 0.01 Aa	0.41 ± 0.11 ABa	0.08 ± 0.01 Aa	0.09 ± 0.03 Aa	12.36 ± 2.92 Aa	12.36 ± 3.79 ABb	6.47 ± 0.87 Aa	4.76 ± 2.34 Aab	81.02 ± 27.96 Aa	52.93 ± 7.17 Aa
D2	A1	3.54 ± 0.11 Ab	3.51 ± 0.36 Ac	0.22 ± 0.07 Ab	0.3 ± 0.06 Aa	0.04 ± 0 Bb	0.02 ± 0 Bb	17.28 ± 4.98 Aa	12.35 ± 4.12 Aab	6.23 ± 1.42 ABa	13.68 ± 4.52 Aa	102.96 ± 10.02 Aab	157.59 ± 21.45 Aa
	A2	3.98 ± 0.21 Ab	3.71 ± 0.25 Abc	0.43 ± 0.18 AB	0.35 ± 0.08 Aa	0.09 ± 0.02 Aab	0.08 ± 0.01 ABa	10.43 ± 3.95 Ba	11.03 ± 2.98 Bb	5 ± 1.51 ABa	4.77 ± 1.79 Ab	48.45 ± 10.82 Ac	49.1 ± 3.72 Ac
	A3	5.3 ± 1.12 Aa	5.04 ± 0.29 ABa	0.52 ± 0.23 Aab	0.41 ± 0.02 Aa	0.1 ± 0.05 Aa	0.06 ± 0.02 Ca	11.47 ± 5.3 ABa	12.32 ± 0.88 Aab	5.38 ± 0.9 Aa	7.42 ± 2.1 Ab	60.76 ± 24.65 Abc	91.75 ± 28.43 Abc
	A4	5.78 ± 0.97 Aa	4.87 ± 1.24 Aab	0.58 ± 0.11 ABa	0.37 ± 0.07 ABa	0.06 ± 0.03 Cab	0.08 ± 0.01 Aa	10.16 ± 1.95 ABa	13.29 ± 2.01 ABab	12.29 ± 7.89 Aa	4.43 ± 0.49 Ab	115.3 ± 49.46 Aa	58.77 ± 9.41 Abc
	A5	5.34 ± 0.16 Aa	5.24 ± 0.79 Aa	0.48 ± 0.23 Aab	0.32 ± 0.03 Ba	0.08 ± 0.02 Aab	0.05 ± 0.02 Aa	12.45 ± 4.43 Aa	16.34 ± 1.3 Aa	6.43 ± 4.51 Aa	6.42 ± 2.25 Ab	66.81 ± 17.12 Aabc	106.57 ± 43.22 Ab
D3	A1	3.64 ± 0.33 Ac	3.49 ± 0.23 Ac	0.37 ± 0.08 Ab	0.27 ± 0.12 Ab	0.05 ± 0.02 Bb	0.05 ± 0.01 Ab	10.36 ± 3.47 Aa	14.65 ± 4.86 Aab	8.47 ± 4.07 ABa	5.27 ± 1.63 Ba	78.58 ± 19.99 Aab	72.76 ± 15.57 Bab
	A2	4.26 ± 0.08 Abc	3.85 ± 0.46 Abc	0.26 ± 0.1 Bb	0.39 ± 0.07 Aab	0.1 ± 0.03 Aa	0.08 ± 0.01 Aa	18.23 ± 6.34 Aa	10.07 ± 2.17 Bb	2.87 ± 1.85 Bb	4.77 ± 1.57 Aa	44.99 ± 11.29 Ac	45.98 ± 6.08 Ac
	A3	5.19 ± 0.94 Aabc	5.24 ± 0.23 Aa	0.27 ± 0.07 Ab	0.41 ± 0.06 Aab	0.06 ± 0.01 Ab	0.1 ± 0.01 ABCa	19.63 ± 5.5 Aa	12.98 ± 1.42 Aab	4.7 ± 1.29 Aab	4.28 ± 0.32 Ba	88.14 ± 10.9 Ba	55.49 ± 6.66 Bbc
	A4	5.63 ± 1.07 Aab	5.18 ± 0.34 Aa	0.36 ± 0.14 Bb	0.3 ± 0.07 Bb	0.08 ± 0.01 BCab	0.06 ± 0.01 Abc	17.66 ± 7.85 Aa	17.74 ± 4.99 Aa	4.43 ± 2.33 Aab	5.09 ± 1.27 Aa	67.61 ± 14.54 ABabc	86.17 ± 4.12 Aa
	A5	5.93 ± 1.18 Aa	4.18 ± 0.22 Ab	0.62 ± 0.19 Aa	0.5 ± 0.04 Aa	0.11 ± 0.03 Aa	0.07 ± 0.01 Aab	9.8 ± 1.2 Aa	8.41 ± 0.26 Bb	5.74 ± 0.94 Aab	6.91 ± 1.88 Aa	56.32 ± 12.38 Abc	57.86 ± 13.8 Abc
D4	A1	3.6 ± 0.27 Ac	3.75 ± 0.34 Ab	0.2 ± 0.01 Ac	0.33 ± 0.07 Aa	0.05 ± 0.01 Ba	0.05 ± 0.01 Abc	18.22 ± 2.15 Aa	11.71 ± 1.75 Aa	3.84 ± 0.73 BbA	6.28 ± 0.15 Ba	70.98 ± 20.72 ABa	73.58 ± 11.13 Ba
	A2	3.95 ± 0.16 Abc	3.95 ± 0.61 Ab	0.38 ± 0.1 ABb	0.43 ± 0.11 Aa	0.07 ± 0.05 Aa	0.03 ± 0.02 Cc	11.06 ± 3.32 ABbc	9.92 ± 3.8 Ba	6.78 ± 3.88 ABab	13.55 ± 13.36 Aa	73.17 ± 39.82 Aa	167.78 ± 195.92 Aa
	A3	5.9 ± 1.65 Aa	4.66 ± 0.33 Bab	0.39 ± 0.05 Ab	0.31 ± 0.05 Aa	0.06 ± 0.01 Aa	0.1 ± 0.02 ABa	15.25 ± 4.19 ABab	15.07 ± 2.45 Aa	6.48 ± 0.68 Aab	3.23 ± 0.98 Ba	100.66 ± 36.22 Aa	47.27 ± 9.59 Ba
	A4	6.02 ± 1.43 Aa	5.23 ± 0.32 Aa	0.7 ± 0.07 ABa	0.41 ± 0.09 ABa	0.07 ± 0.02 Ca	0.07 ± 0.02 Ab	8.86 ± 3.04 Bc	13.16 ± 3.21 ABa	10.08 ± 2.68 Aa	6.18 ± 2.67 Aa	84.25 ± 10.37 ABa	75.76 ± 11.94 Aa
	A5	5.5 ± 0.08 Aab	4.98 ± 0.81 Aa	0.4 ± 0.07 Ab	0.36 ± 0.1 ABa	0.07 ± 0.02 Aa	0.06 ± 0.01 Ab	14.15 ± 3 Aabc	14.68 ± 5.63 Aa	6.02 ± 2.44 Aab	6.13 ± 1.92 Aa	80.33 ± 20.77 Aa	83.06 ± 15.12 a
D5	A1	3.47 ± 0.32 Ac	3.41 ± 0.28 Ac	0.28 ± 0.03 Ab	0.22 ± 0.03 Ac	0.08 ± 0.02 Aab	0.02 ± 0 Bc	12.74 ± 2.63 Aa	15.65 ± 2.65 Aa	3.48 ± 0.4 Ba	10.66 ± 1.96 Aa	45.03 ± 14.52 Ba	163.51 ± 11.56 Aa
	A2	4.29 ± 0.15 Abc	3.92 ± 0.36 Abc	0.33 ± 0.06 ABb	0.35 ± 0.1 Abc	0.07 ± 0.01 Ab	0.05 ± 0.02 BCbc	13.28 ± 2.11 ABa	11.63 ± 3.06 Bab	4.45 ± 0.6 Ba	8.88 ± 6.56 Aa	58.51 ± 7.3 Aa	89.91 ± 38.07 Aabc
	A3	5.53 ± 1.26 Aab	4.78 ± 0.24 ABab	0.59 ± 0.26 Aab	0.39 ± 0.06 Aab	0.07 ± 0.01 Ab	0.12 ± 0.03 Aa	10.31 ± 3.25 Ba	12.47 ± 2.21 Aab	8.05 ± 2.98 Aa	3.36 ± 1.13 Ba	76.58 ± 10.4 Aa	40.19 ± 7.84 Bc
	A4	5.6 ± 1.05 Aab	5.43 ± 0.05 Aa	0.73 ± 0.3 Aa	0.51 ± 0.02 Aa	0.12 ± 0.02 ABa	0.09 ± 0.04 Aab	8.54 ± 3.36 Ba	10.56 ± 0.26 Bb	6.61 ± 4.22 Aa	7.01 ± 3.01 Aa	47.22 ± 8.84 Ba	73.86 ± 31.83 Abc
	A5	6.97 ± 1.06 Aa	5.43 ± 1.24 Aa	0.52 ± 0.15 Aab	0.42 ± 0.12 ABab	0.1 ± 0.04 Aab	0.05 ± 0.03 Abc	14.5 ± 5.73 Aa	13.21 ± 0.77 ABab	5.66 ± 3.23 Aa	9.99 ± 6.95 Aa	77.14 ± 34.61 Aa	128.39 ± 83.25 Aab

Note: For all measured variables, A represents fertilization application rate and D represents organic fertilizer mixing proportion. Values represent means ± SE (*n* = 4). Capital letters mark significant differences among various organic fertilizer proportions under an identical fertilization rate, whereas lowercase letters denote significant differences across fertilization rates for the same organic fertilizer proportion.

## Data Availability

The original contributions presented in this study are included in the article. Further inquiries can be directed to the corresponding author.

## Supplementary Materials

The following supporting information can be downloaded at https://www.mdpi.com/article/10.3390/biology15191780/s1: Figure S1: Plot layout diagram. Different colors represent different blocks; Figure S2: Effects of organic fertilizer addition on fungal Shannon index; Figure S3: LEfSe analysis of differential fungal taxa under organic fertilizer addition; Figure S4: Effects of organic fertilizer addition on soil bacterial Shannon index; Figure S5: LEfSe analysis of differential bacterial taxa under organic fertilizer addition; Table S1: Partial η^2^ values of aboveground dry matter yield affected by organic fertilizer proportion, fertilization rate and their interaction.
