# Supplementary material for: Effects of Combined Peat Powder and Fermented Manure on Oat Yield, Soil Enzyme Activities and Microbial Community Composition in Horqin Sandy Land
Source: Biology (Basel). 2026 Oct 8;15(19):1780. doi: 10.3390/biology15191780 (PMC13664378; doi:10.3390/biology15191780)
Supplement: Supplementary file 1 [file biology-15-01780-s001.zip › biology-4562046-supplementary.pdf]

Supplementary information for

Effects of Combined Organic Fertilizer on Oat Yield, Microbial Diversity and Soil Nutrient in Sandy Agro-pastoral Ecotone

Ruozhuang Zhao †, Hai-Shuang Liu †, Jiale Na, Tao Li,Ziwei Li, Tiexia Zhu, Zhaoxu Nie, and Kai Gao [*]

College of Grassland, Inner Mongolia Minzu University, Tongliao 028000, China

† These authors contributed equally to this work and should be considered co-first authors.

* Correspondence:gaokai555@126.com

D1A1	D4A5	D5A4	D1A4	D5A2	D1A1	D4A5	D5A4	D1A4	D5A2
D2A2	D3A2	D5A3	D4A3	D2A4	D2A2	D3A2	D5A3	D4A3	D2A4
D3A3	D1A3	D5A5	D2A5	D2A3	D3A3	D1A3	D5A5	D2A5	D2A3
D4A4	D3A4	D1A2	D4A2	D4A1	D4A4	D3A4	D1A2	D4A2	D4A1
D2A1	D5A1	D3A1	D1A5	D3A5	D2A1	D5A1	D3A1	D1A5	D3A5
D1A1	D4A5	D5A4	D1A4	D5A2	D1A1	D4A5	D5A4	D1A4	D5A2
D2A2	D3A2	D5A3	D4A3	D2A4	D2A2	D3A2	D5A3	D4A3	D2A4
D3A3	D1A3	D5A5	D2A5	D2A3	D3A3	D1A3	D5A5	D2A5	D2A3
D4A4	D3A4	D1A2	D4A2	D4A1	D4A4	D3A4	D1A2	D4A2	D4A1
D2A1	D5A1	D3A1	D1A5	D3A5	D2A1	D5A1	D3A1	D1A5	D3A5

D fertilizer proportions
A fertilization rates

4m

5m

Figure S1. Plot layout diagram.Different colors represent different blocks, with a 50-cm-wide aisle between plots. Each plot is 4 m × 5 m in size.

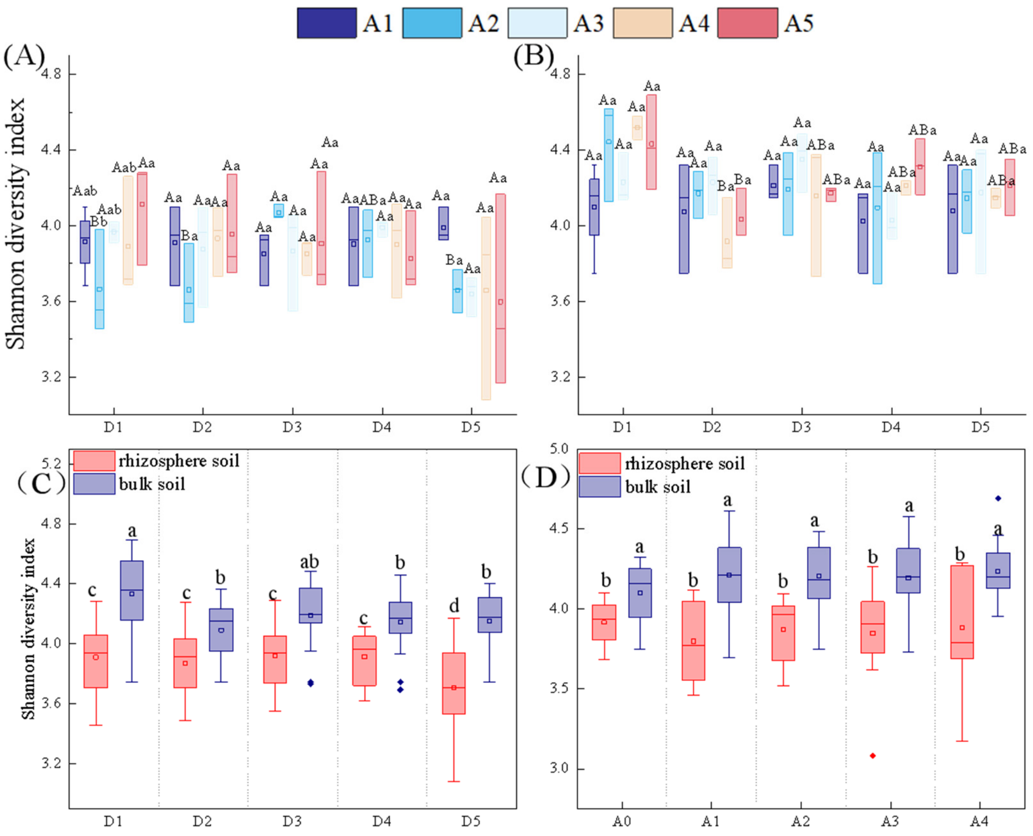

Figure S2. Effects of organic fertilizer addition on fungal Shannon index. effect of fertilization proportions and rates on fungal Shannon index in the rhizosphere soil (A) or bulk soil (B); effect of fertilization proportions (C) or fertilization rates (D) on fungal Shannon index in the rhizosphere soil and bulk soil. Values represent means ± SE (n = 4). Capital letters mark significant differences among various organic fertilizer proportions under an identical fertilization rate, whereas lowercase letters denote significant differences across fertilization rates for the same organic fertilizer proportion.

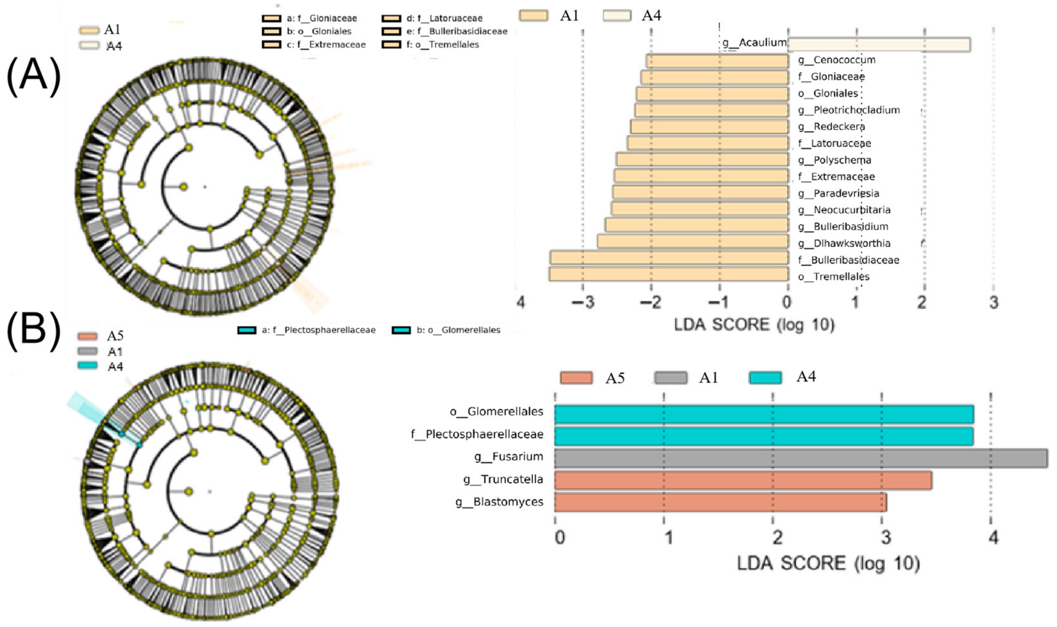

Figure S3. LEfSe analysis of differential fungal taxa under organic fertilizer addition. Organic fertilization rates in rhizosphere soil (A) and bulk soil (B). The left panel is evolutionary branching diagram of soil fungi, and the right panel is LDA histogram. LDA threshold set to 2.0.

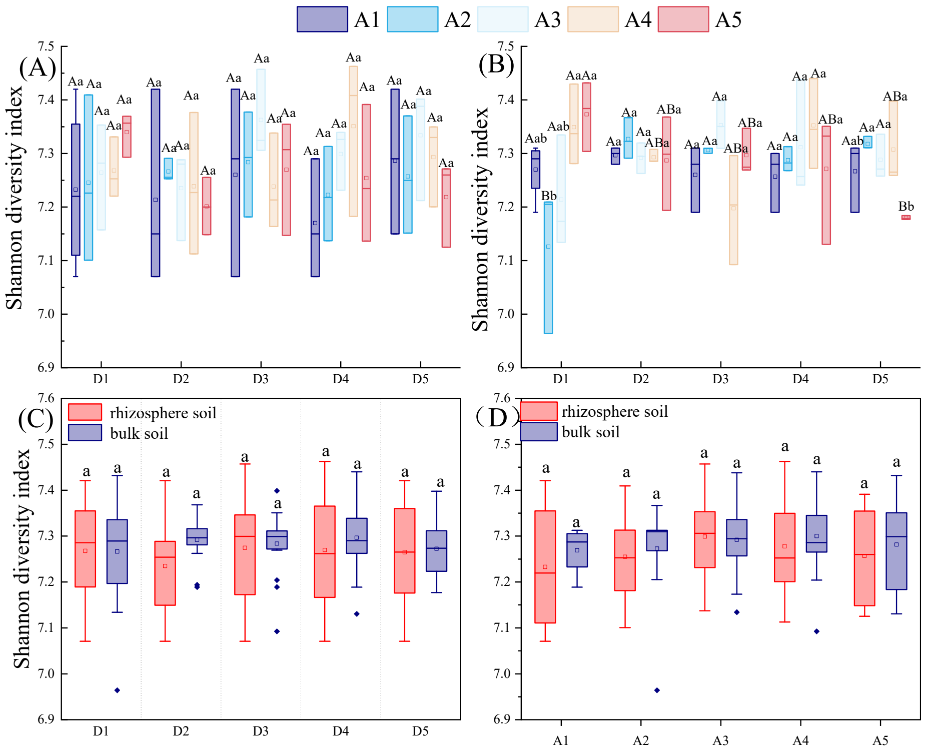

Figure S4. Effects of organic fertilizer addition on soil bacterial Shannon index. effect of fertilization proportions and rates on bacterial Shannon index in the rhizosphere soil (A) or bulk soil (B); effect of fertilization proportions (C) or fertilization rates (D) on bacterial Shannon index in the rhizosphere soil and bulk soil.Values represent means ± SE (n = 4).

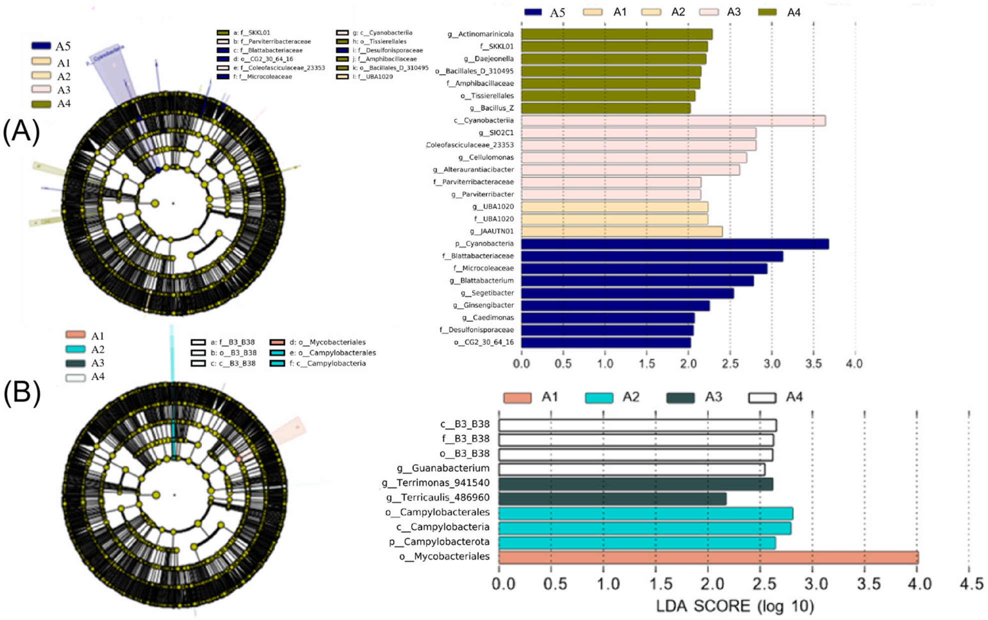

Figure S5. LEfSe analysis of differential bacterial taxa under organic fertilizer addition. (A) in rhizosphere soil; (B) fertilization rates proportions in bulk soil. The left panel is evolutionary branching diagram of soil fungi, and the right panel is LDA histogram. LDA threshold set to 2.0.

Table S1 Partial η^2 values of aboveground dry matter yield affected by organic fertilizer proportion, fertilization rate and their interaction

	partial eta squared
Genus	aboveground dry matter yield
D	0.650
A	0.973
D*A	0.856
